# Analgesic treatment limits surrogate parameters for early stress and pain response after experimental subarachnoid hemorrhage

**DOI:** 10.1186/s12868-019-0531-7

**Published:** 2019-09-18

**Authors:** Irina Staib-Lasarzik, Nadine Nagel, Anne Sebastiani, Eva-Verena Griemert, Serge C. Thal

**Affiliations:** Department of Anesthesiology, University Medical Center of the Johannes Gutenberg-University, Langenbeckstrasse 1, 55131 Mainz, Germany

**Keywords:** Pain, Analgesia, Subarachnoid hemorrhage, Traumatic brain injury, Perioperative analgesia, Buprenorphine, Carprofen

## Abstract

**Background:**

In animal research, authorities require a classification of anticipated pain levels and a perioperative analgesia protocol prior to approval of the experiments. However, data on this topic is rare and so is the reported use of analgesics. We determined surrogate parameters of pain and general well-being after subarachnoid hemorrhage (SAH), as well as the potential for improvement by different systemic analgesia paradigms. Brain injury was induced by filament perforation to mimic SAH. Sham-operated mice were included as surgical control groups with either neck or no-neck preparation. Mice with controlled cortical impact (CCI) injury were included as a control group with traumatic brain injury (TBI), but without neck preparation. Mice were randomized to buprenorphine, carprofen, meloxicam, or vehicle treatment. 24 h after SAH, CCI or sham surgery, pain and stress levels were assessed with a visual assessment score and the amount of food intake was recorded.

**Results:**

Neck preparation, which is required to expose the surgical field for SAH induction, already increased pain/stress levels and sham surgeries for both CCI and SAH reduced food intake. Pain/stress levels were higher and food intake was lower after SAH compared with CCI. Pain/stress levels after CCI without analgesic treatment were similar to levels after SAH sham surgery. Pain treatment with buprenorphine was effective to reduce pain after SAH, whereas lower pain/stress intensity levels after CCI were not improved.

**Conclusion:**

This study emphasizes the importance of pain and stress assessment after surgeries and the efficacy of buprenorphine to improve pain and comfort levels after experimental SAH.

## Background

Pain after invasive experimental procedures is a common problem in animal research. For appropriate handling and care of laboratory animals during experimental scientific procedures the use of perioperative analgesics is essential. However, in a structured literature review the number of reported administration of systemic analgesics was 20%, indicating that the majority of animals are still withheld from perioperative analgesic treatment [1]. In the field of brain research, most mouse models require a surgical procedure to induce brain injury, such as the subarachnoid hemorrhage (SAH) or the traumatic brain injury (TBI) models. A review of the literature showed that these procedures are often performed without mentioning perioperative analgesia, e.g. SAH [2–5] and TBI [6–13]. Only a few studies report the administration of perioperative analgesics in experimental SAH [14, 15]. This is even more surprising as literature calls for refined pain assessment in SAH and TBI models [16].

Information on pain and stress induced by experimental models and data on effective measures to prevent pain and stress are required by legislation and ethical authorities prior to approval of experiments. There are few recommendations on postoperative analgesia for laboratory animals such as the recommendations of the German Society for laboratory animal science (GV-Solas [17]); however, these are not adapted to the respective experimental models.

The purpose of the present study was to provide missing data in a commonly used model of experimental SAH in mice. The study was designed to quantify perioperative pain and stress levels and to determine the best choice of three recommended systemic analgesic paradigms. For a better comparability perioperative pain and stress levels were also determined after isolated brain injury by controlled cortical impact (CCI). Sham-operated mice were included as control groups for stress by the surgical procedure itself.

## Methods

All experiments were approved by the local Animal Ethics Committee (Landesuntersuchungsamt Rheinland-Pfalz) and performed in accordance with the German animal protection law. A total of 87 male C57Bl/6N mice (weight 18–23 g, Charles River Laboratories, Sulzfeld, Germany) were included in the study.

### Experimental groups

#### SAH groups

Mice were randomized to the following groups: treatment with buprenorphine (BUP, n = 8), carprofen (CAR, n = 8), meloxicam (MEL, n = 8), vehicle (VEH, n = 8), or sham-operation without analgesic treatment (SHAM, n = 5).

#### CCI groups

Mice were randomized to the following groups (n = 10/group): treatment with buprenorphine (BUP), carprofen (CAR), meloxicam (MEL), vehicle (VEH), or sham-operation without analgesic treatment (SHAM).

SHAM groups without analgesic treatment were included to determine the pain levels that are solely related to the surgical procedures and not due to the experimentally induced brain pathologies.

### Drug administration

Buprenorphin (Temgesic^®^, Essex, Munich, Germany) was diluted in 0.9% NaCl to the concentration 0.01 mg/ml and administered in a dosage of 0.1 mg/kg subcutaneously (s.c.). Carprofen (Rimadyl^®^, Pfizer GmbH, Berlin, Germany) was diluted in 0.9% NaCl to the concentration 0.5 mg/ml and s.c. administered in a dosage of 5 mg/kg. Meloxicam (Metacam^®^, Boehringer Ingelheim, Germany) was diluted in 0.9% NaCl to the concentration 0.1 mg/ml and s.c. administered in a dosage of 1 mg/kg. Injection volume was dependent on daily body weight and 0.1 ml of each solution was injected for every 10 g body weight. The first injection was administered during anesthesia prior to the beginning of surgery. Buprenorphine and carprofen injections were repeated after 12 h. Animals with meloxicam and vehicle were administered a vehicle injection (0.9% NaCl) after 12 h. The applied dosages were based on the recommendations of the German Society for laboratory animal science (GV-Solas [17]).

### Animal preparation

Anesthesia was induced in a bell jar filled with 4% isoflurane (Abbott, Wiesbaden, Germany) and maintained by inhalation via face-mask (1.4–2% isoflurane in 40% O_2_ and 60% N_2_). A thermostatically regulated, feedback-controlled heating pad was used to maintain body temperature at 37 °C (Hugo Sachs, March-Hugstetten, Germany). After surgery, animals were placed in a neonatal incubator (IC8000, Draeger, Luebeck, Germany) for 1 h with controlled air temperature (33 °C) and ambient humidity (35%) to maintain a constant body temperature.

### Subarachnoid hemorrhage

The subarachnoid hemorrhage model was performed as previously described [18]. In brief, the neck was opened by a midline incision and the left carotid artery was exposed by surgical preparation. A 5-0 monofilament was advanced via the external carotid artery into the internal carotid artery until the ipsilateral cerebral blood flow (CBF) decreased to ensure the position of the tip of the filament near the bifurcation of the internal carotid artery and the middle cerebral artery. Then the filament was pushed forward until a sudden increase of the intracranial pressure (ICP) indicated successful induction of SAH. Subsequently, the suture was withdrawn into the external carotid artery to allow full perfusion of the internal carotid artery. For monitoring of ICP, a Codman ICP microsensor (Johnson & Johnson Medical GmbH, Norderstedt, Germany) was placed in the epidural space of the right hemisphere. A flexible laser-Doppler probe (Periflux 4001, Perimed, Järfälla, Sweden) was glued onto the skull above the territory of the left middle cerebral artery for assessment of regional cerebral blood flow. Both probes were removed after surgery.

### Subarachnoid hemorrhage—sham operation

In sham operated animals, the same surgical procedure was performed including the preparation of the left carotid artery. However, no intracranial bleeding was induced by advancement of filament and no ICP probe was placed. In detail, a 1 cm midline incision was made between the manubrium and the jaw. The submandibular glands were bluntly divided to expose the surgical field beneath. The carotid artery was separated from the vagus nerve. The bifurcation of the common carotid artery into the left external carotid artery (ECA) and left internal carotid artery (ICA) was identified, and the ECA and ICA were isolated from surrounding nerves and fascia. The ECA was then ligated. Great care was taken to minimize damage to trachea, sternocleidomastoid muscle and the small nerve fibers that are adjacent to the carotid artery. Afterwards, the skin was carefully closed, isoflurane discontinued, and the animals transferred to their cages.

### Traumatic brain injury

The brain trauma model was performed as previously described [19]. The skull was fixed in a stereotactic frame (Kopf Instruments, Tujunga, USA) and a craniotomy was performed above the right parietal cortex between the sagittal, lambdoid, and coronal sutures, and the insertion of the temporal muscle with a saline cooled high-speed drill. The lesion was induced perpendicular to the surface of the brain with a custom fabricated pneumatic controlled cortical impactor device (L. Kopacz, Mainz, Germany; diameter of the impactor tip: 3 mm; impact velocity: 8 m/s; impact duration: 150 ms; displacement: 1 mm). The craniotomy was closed with the initially removed bone flap using conventional tissue glue (Histoacryl^®^, Braun-Melsungen, Germany). The skin was carefully closed, isoflurane discontinued, and the animals transferred to their cages.

### Traumatic brain injury—sham operation

In sham operated animals, the same surgical procedure was performed including the preparation of the skull and removal of the bone flap. In detail, the skull was fixed in a stereotactic frame (Kopf Instruments, Tujunga, USA). The skin was opened with a 1.5 cm midline incision. A craniotomy was performed above the right parietal cortex between the sagittal, lambdoid, and coronal sutures, and the insertion of the temporal muscle with a saline cooled high-speed drill. Afterwards the bone flap was remove. The craniotomy was closed with the removed bone flap and fixed with conventional tissue glue (Histoacryl^®^, Braun-Melsungen, Germany). The skin was carefully closed, isoflurane discontinued, and the animals transferred to their cages.

### Pain assessment

Body weight was measured before surgery and before euthanasia. Food intake was measured 24 h preoperatively and 24 h postoperatively. For assessment of food intake, three pre-dried food pellets were weighed before placement in the cage and after 24 h. The remaining weight of the pre-dried uneaten pellets was subtracted from the initial weight. For better comparison, food intake was calculated per 1 g body weight (daily food intake/actual body weight).

Pain intensity was assessed with the visual assessment score adapted from Adamson et al. (Table 1) [20]. Points were awarded for abnormal animal behavior (best 0 points, worst 29 points). Pain levels were assessed directly before surgery and 24 h after the end of surgery directly before euthanasia, which means 12 h after the last pain treatment with buprenorphine and carprofen or 24 h after the last pain treatment with meloxicam. Scoring was performed by an experienced investigator blinded to the group allocation and experienced in the use of the visual assessment score after brain trauma in mice. After performance of the scoring task animals were killed by cervical dislocation after 1-min exposure to 4 % isoflurane.

**Table 1:** Criteria for the visual assessment score, points are awarded for abnormal animal behavior (best 0 points, worst 29 points)

Item	Points	Best	Worst
Nest building	(0) Normal	0	2
	(1) Use of paper towel		
	(2) No use of paper towel		
Teeth grinding	(2) If present	0	2
Vocalization	(2) If present	0	2
Hair coat	(0) Normal	0	6
	(2) Not well groomed		
	(4) Rough, dirty		
	(6) Very rough, dirty		
Eyes	(0) Open, alert	0	4
	(2) Squinted		
	(4) Closed		
Coordination and posture	(0) Normal	0	6
	(1) Lightly hunched		
	(2) Walks hunched		
	(3) Walks hunched and slowly		
	(4) No running		
	(5) Hunched, strumbles		
	(6) Hunched, no movement		
Overall condition	(0) Normal	0	3
	(1) Rough, acts normal		
	(2) Rough, depressed		
	(3) Very rough, very depressed		
Withering	(0) Skin fold < 1 s	0	4
	(2) Skin fold > 1 s		
	(4) Skin fold > 2 s		
Total		0	29

### Statistical analysis

Statistical analysis was performed with GraphPad Prism 8 (GraphPad Software, La Jolla, CA). Intracranial pressure at baseline and peak ICP levels were analyzed with one-way ANOVA and Holm–Šidák’s multiple comparison test. Visual assessment score data are presented as box plots and were compared with Kruskal–Wallis-Test and Dunn’s multiple comparisons test for post hoc comparisons. Power analyses yielded a power of 89% for the SAH groups and 96% for the CCI groups regarding differences in the pain assessment score. Food intake data are presented as mean ± SD and were analyzed with one-way ANOVA before and after brain injury. Due to the limited power in small samples, we did not perform formal goodness-of-fit tests prior to the ANOVA, but instead relied on the graphical assessment of distribution characteristics [21]. Normality was checked by inspecting the unimodality and symmetry of histograms and standard deviations. For post hoc comparisons, Holm–Šidák’s multiple comparison test was employed. Difference between before and after brain injury were determined between the SHAM groups with the Welch’s t-test. Differences were considered significant at the P < 0.05 level.

## Results

### Mortality

All mice of the CCI and sham groups survived and completed the study protocol. In the SAH groups, three animals of each group (with the exception of sham-operated animals) died within 24 h due to seriously weakened overall conditions. These mice were excluded from the study protocol.

### Intracranial pressure after SAH

Intracranial pressure was continuously monitored with the ICP probe before, during, and after the induction of SAH. Baseline values did not differ between groups (VEH 20.2 ± 9.1 mmHg, BUP 13.2 ± 3.1 mmHg, CAR 14.8 ± 5.8 mmHg, MEL 13.8 ± 5.0 mmHg). Peak values did not differ between groups as well (VEH 54.4 ± 17.8 mmHg, BUP 53.6 ± 19.6 mmHg, CAR 48.2 ± 18.0 mmHg, MEL 69.8 ± 19.8 mmHg).

### Pain and stress levels

**Fig. 1:**
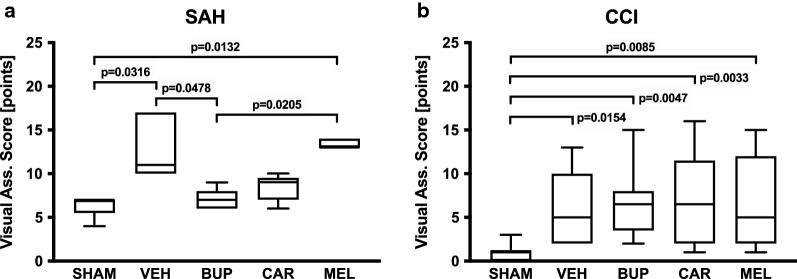
Visual assessment score (best 0 points, worst 29 points) as an indicator of pain intensity after subarachnoid hemorrhage (SAH, **a**) and controlled cortical impact (CCI, **b**). *SAH* subarachnoid hemorrhage, *CCI* controlled cortical impact, *SHAM* sham operation, *VEH* vehicle solution, *BUP* buprenorphine, *CAR* carprofen, *MEL* meloxicam (Kruskal–Wallis test and Dunn’s multiple comparison test; SAH: N = 25, H = 18.67, P = 0.0009; CCI: N = 50, H = 18.69, P = 0.0009)

The visual assessment score was determined as indicator for perioperative pain and stress intensity (Fig. 1a, b). Visual assessment score levels significantly increased after performance of SAH compared with sham-operated animals (VEH 13.0 ± 3.7 vs. SHAM 6.4 ± 1.3 points, P = 0.0316, n = 5 each, Kruskal–Wallis test: N = 25, H = 18.67, P = 0.0009). Similar to SAH, visual assessment score levels after CCI demonstrated significant differences between CCI and sham-operated animals (VEH 5.9 ± 4.2 vs. SHAM 0.9 ± 1.0 points, P = 0.0154, n = 10 each, Kruskal–Wallis test: N = 50, H = 18.69, P = 0.0009). Visual assessment score levels differed substantially in respect that absolute visual assessment score levels after CCI (VEH) were similar to scores after SAH sham surgery. After SAH, only BUP (7.0 ± 1.2 points, P = 0.0478) significantly reduced distress compared with VEH and compared with MEL treatment (13.4 ± 0.55 points, P = 0.0205), whereas all treatment protocols failed to influence the rather low visual assessment score levels after CCI.

### Food intake

**Fig. 2:**
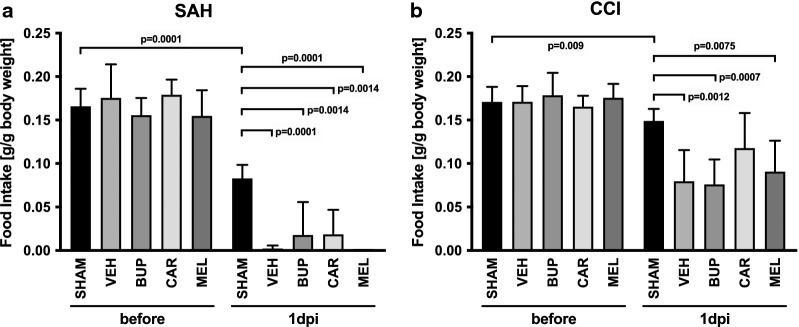
Food intake after subarachnoid hemorrhage (SAH, **a**) and controlled cortical impact (CCI, **b**). *SAH* subarachnoid hemorrhage, *CCI* controlled cortical impact, *SHAM* sham operation, *VEH* vehicle solution, *BUP* buprenorphine, *CAR* carprofen, *MEL* meloxicam, *g* gramm, *dpi* day post injury (one-way ANOVA and Holm–Šidák’s multiple comparison test; **a** before SAH: F = 0.8336, P = 0.5205; after SAH: F = 11.27, P < 0.0001; **b** before CCI: F = 0.7304, P = 0.5759; after CCI: F = 7.221, P = 0.0002; data are presented as mean ± SD)

Food intake (Fig. 2a, b) was quantified and demonstrated a substantial reduction in the amount of eaten food after both SAH and CCI. SAH resulted in an almost cessation of food intake (VEH 0.002 ± 0.004 vs. SHAM 0.083 ± 0.016 g/g body weight, P = 0.0001, one-way ANOVA: F = 11.27, P < 0.0001), which was not significantly influenced by pain treatment. Food intake was reduced to a lower extent after CCI (VEH 0.080 ± 0.036 vs. SHAM 0.149 ± 0.014 g/g body weight, P = 0.0012, one-way ANOVA: F = 7.221, P = 0.0002). Sham-operation also significantly reduced food intake both after SAH-SHAM (before: 0.166 ± 0.02 vs. after: 0.083 ± 0.016 g/g body weight, P = 0.0001, Welch’s t-test: F = 1.677, P = 0.6289) and after CCI-SHAM surgery (before: 0.171 ± 0.017 vs. after: 0.149 ± 0.014 g/g body weight, P = 0.009, Welch’s t-test: F = 1.552, P = 0.5753). In all groups, pain treatment did not influence food intake.

## Discussion

We quantified surrogate parameters of perioperative pain and stress in a commonly used SAH model and compared the efficacy of three analgesic paradigms. Pain and stress levels were assessed with the visual assessment score and levels after SAH were significantly improved by treatment with buprenorphine. In summary, the data show the need for postoperative analgesics for the prevention and treatment of pain and distress after experimental SAH.

According to the animal protection law and as outlined in e.g. the European guidelines, it is required to classify the anticipated pain levels and to provide adequate analgesic treatment in experimental animal research. However, data on this topic is rare and so is the reported use of analgesics. Following the report of Stokes et al. [1] no significant steps have been taken to improve acute postoperative pain in experimental brain research, leading to the call by Pinkernell et al. [16] for mandatory analgesia after SAH and TBI in rodents. Although analgesic treatment may be standard for some general surgical procedures, there is still great concern in the neuroscience community about the confounding effects of analgesics on outcome after experimental brain injury.

In order to allow a simple quantification of pain and stress, the visual assessment score was selected as surrogate parameter and was adapted from Adamson et al. [20]. The score was modified by adding parameters (nest building, teeth grinding, vocalization, and assessment of the skin fold) to increase the sensitivity. The visual assessment score was performed before surgery and after 24 h. Studies assessing pain in mice often note that scores are not assessed at night when mice are most likely to show signs of pain [20] and that only a limited range of behavior is determined for a short amount of time [22]. A more sensitive methods for the detection of pain in mice may be the “HomeCageScan”, an automated behavior recognition software [22], or the Mouse Grimace Scale [23]. However, implementation of these techniques is complex, time consuming and not widely established.

SAH and CCI may also cause alteration of pain perception, such as allodynia or hyperalgesia. There are several tests for the assessment of allodynia and hyperalgesia, such as the Frey filament test [24]; however, the purpose of this study was to assess the pain due to the surgical preparation and the pain of the brain injury itself induced by subarachnoid hemorrhage and traumatic brain injury—and not nociception.

In the present investigation, SAH caused high pain/stress levels, which was improved by pain treatment. Therefore, analgesics should routinely be administered after SAH in mice. In addition, surgical incision of the neck, preparation of the carotid artery, and the preparation of the temporal muscle are accused to contribute to the pain load. This is supported by the present study, which shows marked differences in visual assessment score levels between SAH-sham surgery and CCI-sham surgery. The data suggest that the surgical preparation of the neck and the carotid artery causes a higher stress burden compared to surgery to the skull only—as required for the CCI model. Unfortunately, the study did not include pain treatment of sham animals. Therefore, we do not know if stress/pain caused by SAH-sham surgery can be improved by the tested pain medication.

Pain and stress levels after SAH were significantly improved by administration of buprenorphine, but not after treatment with meloxicam or carprofen. This finding is in line with results of a recent study that attempted to reduce pain using e.g. meloxicam after laparotomy in mice [22]. Meloxicam in dosages of 1 mg/kg, 5 mg/kg, or 20 mg/kg did not improve pain levels assessed with body weight, automated behavior analyses, and Mouse Grimace Scale evaluating changes in eyes, ears, nose, cheeks, and whiskers. In the present study, meloxicam was administered in a dosage of 1 mg/kg before surgery. Although this dosage was selected based on the recommendations of the German association for laboratory animals (GV-Solas), the dosage may have been too low for a significant pain reduction. Similarly, carprofen could have been effective in higher dosages to limit pain and stress after SAH. Unfortunately, the study was designed to test established protocols and not to determine the most effective drug dose. Based on the present data, we therefore cannot predict a potential dose-dependent increase in analgesic action.

In contrast to SAH, all three analgesic treatments failed to influence pain/stress levels after traumatic brain injury. CCI-sham operated animals showed only a small increase in visual assessment score levels, and CCI animals showed similar pain/stress scores compared to SAH-sham animals. Taken together, the data suggest that the skull preparation alone does not cause relevant pain, whereas CCI induction increase pain/stress to a similar intensity as in SAH-sham animals. Due to lack of different tested dosages we cannot rule out that higher dosages might have a beneficial impact on pain/stress levels after brain trauma. The present data suggest that CCI animals do not benefit from a pain therapy.

Although pain and stress levels after SAH were improved by buprenorphine, food intake did not increase to sham levels. A possible explanation is that buprenorphine, like other opioids, impairs the gastrointestinal system—the most common symptom being nausea [25]. Another possible explanation is the route of application of the analgesics. Subcutaneous injections were performed every 12 h. Frequent injections are known to cause loss in body weight even in the absence of a surgery due to stress response [26]. However, the awake animals were injected only once. We therefore think that stress due to the injection plays only a subordinate role in this study. To reduce stress in future studies, a possible alternative is the use of sustained release formulations of buprenorphine and carprofen [27, 28], which may provide superior treatment paradigms with fewer injections. A further alternative route of drug administration is the oral self-administration of buprenorphine in jelly [29]; however, this may not be suitable for mice after SAH, which show an almost cessation of food intake.

An important limitation of our investigation is that we only focused on the early symptoms within the first 24 h after cerebral injury. We therefore cannot give information on the optimal duration of analgesic therapy. The present study determined surrogate parameters of pain and stress, not direct measures such as blood cortisol or catecholamine levels. Future studies are therefore required to investigate optimal drug dosages, treatment duration and the influence of pain treatment on blood catecholamine and cortisol levels.

## Conclusions

We demonstrate that experimental SAH produces higher pain surrogate levels than SAH sham-surgery and CCI, which is significantly improved by buprenorphine treatment. The present data therefore suggest that SAH mice benefit strongly from pain treatment. In contrast to SAH, analgesic treatment did not improve stress and pain levels in animals subjected to experimental TBI.

These observations may seem trivial; however, the results of the present investigation are the first description of post-interventional pain/stress levels in the widely used filament SAH model and after controlled cortical impact injury. The data may provide useful information to estimate the postoperative pain levels and the requirement for analgesic treatment in these experimental models.

## Data Availability

All datasets generated and analyzed during this study are kept in the Dept. of Anesthesiology, Medical Center of the Johannes Gutenberg-University and are available from the corresponding author upon reasonable request.

## Abbreviations

SAH: subarachnoid hemorrhage
CCI: controlled cortical impact
TBI: traumatic brain injury
SHAM: sham operation
VEH: vehicle
BUP: buprenorphine
CAR: carprofen
MEL: meloxicam
ICP: intracranial pressure
